# A New Drug Release Method in Early Development of Transdermal Drug Delivery Systems

**DOI:** 10.1155/2012/953140

**Published:** 2012-08-05

**Authors:** Bing Cai, Karin Söderkvist, Håkan Engqvist, Susanne Bredenberg

**Affiliations:** ^1^Division for Applied Materials Science, Department of Engineering Sciences, The Ångström Laboratory, Uppsala University, Box 534, 751 21 Uppsala, Sweden; ^2^Department of Analytical Chemistry, Orexo AB, P.O. Box 303, 751 05 Uppsala, Sweden

## Abstract

*In vitro* drug release tests are a widely used tool to measure the variance between transdermal product performances and required by many authorities. However, the result cannot provide a good estimation of the *in vivo* drug release. In the present work, a new method for measuring drug release from patches has been explored and compared with the conventional USP apparatus 2 and 5 methods. Durogesic patches, here used as a model patch, were placed on synthetic skin simulator and three moisture levels (29, 57, 198 **μ**L cm^−2^) were evaluated. The synthetic skin simulators were collected after 1, 2, 3, 4, 6, and 24 hours and extracted with pH 1.0 hydrochloric acid solution. The drug concentrations in the extractions were measured by isocratic reverse phase high-pressure liquid chromatography. The results showed that, with the increasing moisture level on the synthetic skin simulator, the drug release rate increased. In comparison with the conventional USP method, the drug release results performed by the new method were in more correlation to the release rate claimed in the product label. This new method could help to differentiate the drug release rates among assorted formulations of transdermal drug delivery systems in the early stage of development.

## 1. Introduction

Transdermal patches delivers drug across skin to the circulatory system to achieve therapeutic effects [1] or to provide local effect of drugs. As one of the fastest growing drug administration routes [2], it has several advantages compared to other traditional delivery methods: controlled release rate, more stable plasma concentration, noninvasive administration, less frequent dosing, and simple application without professional medical aids [3]. Yet, a common problem with transdermal delivery systems is permeation across stratum corneum, which limits the size and properties of drug molecule to pass through. Hence, in addition to the delivery device, skin also serves as another rate-limiting step for many transdermal delivery systems [2, 4–6]. In general, the flux across the skin is dependent upon the hydration of the skin, partitioning, and transport across the stratum corneum and the concentration gradient across the skin [7]. *In vitro* drug release test is an important characterization tool to evaluate the performance of transdermal drug delivery systems (TDDSs). Since this type of test is less costly and easier to implement than *in vivo* studies [8], it is widely used for many purposes during drug development, especially for screening processes and stability assessment of new formulations [9]. USP apparatus 5 (paddle over disc), USP apparatus 6 (rotating cylinder), and USP apparatus 7 (reciprocating holder) are the recommended *in vitro* release testing method for transdermal delivery systems by the authorities [8, 10], but USP apparatus 2 has also been used in some *in vitro* studies of transdermal patches [11, 12]. However, the results of these USP methods for some TDDS were found to be difficult to correlate with the *in vivo* performances, especially for the patches that depend on the skin resistance as the dominating factor for the controlled release [13, 14]. USP methods are, therefore, more useful in quality control processes of TDDS, and permeation tests are commonly used alternatives to give more comparable release profile as *in vivo* result in transdermal formulation development. Franz diffusion cell, horizontal-type permeation systems, and flow-through diffusion cells are some well-known examples [15, 16]. In these methods, a skin or synthetic membrane is placed between the patch and a buffer, simulating the resistance and penetration effects [17]. Several studies have shown that hydration of skin and variation of skin or synthetic membrane could influence the accuracy of the method [7, 18–20]. Hence, an easy-implemented testing method, which could imitate resistance and penetration effect as skin and test under limited moisture conditions, could be helpful in the development of new formulations of TDDS. 

Durogesic was used as the model test patch in this study. It is a fentanyl controlled-release transdermal patch, which is used for moderate to severe chronic pain relief. There are four strengths of the dose on the market and their stated release rate is claimed as 2.38 *μ*g cm^−2 ^h^−1^ for 72 hours [21]. The strength of the dose is proportional to the contact area of the patch. Previous studies have found that the transdermal delivery of fentanyl cause a depot in upper layers of the skin [22, 23]. The depot accumulation of the drug causes delay of drug delivery to the systemic circulation [24, 25]. This indicates that the diffusion and penetration across skin are the primary rate-limiting step of the fentanyl patches.

The purpose of this study was to develop a selective and easy-to-handle test method that could imitate the drug diffusion and moisture level of skin. The aim was that the method should provide a good indication of the *in vivo* performances and be helpful in screening different TDDS formulations from their release rate in the early stage of development. 

## 2. Materials and Methods

### 2.1. Materials

The commercial matrix-type transdermal patch, Durogesic patches (Janssen-Cilag, Belgium) with two strengths, 12 and 75 *μ*g/hr, was used as model patch. Fentanyl base (MacFarlan and Smith, Edinburgh, UK) was a gift from Orexo AB, Sweden. Dish sponge cloth (Wettex) is produced by the Freudenberg group (Norrköping, Sweden). The cloth is made of 35% cotton and 65% cellulose with 2 mm in thickness. In this study it served as a reservoir to collect drug and a synthetic skin simulator (SSS) to mimic the resistance and penetration effect. Its true density and porosity were measured by gas pycnometer and the values were 2.0543 g cm^−3^ and 93.7%, respectively. Reagent grade of sodium hydroxide (NaOH), 37% fuming hydrochloric acid (HCl) and monopotassium phosphate (KH_2_PO_4_) were purchased from Sigma-Aldrich (Stockholm, Sweden). Phosphate buffer solution was made of NaOH and KH_2_PO_4_ and its pH was adjusted to 6.8 ± 0.05.

### 2.2. Drug Release Test of Durogesic Patch on SSS

**Figure 1:**
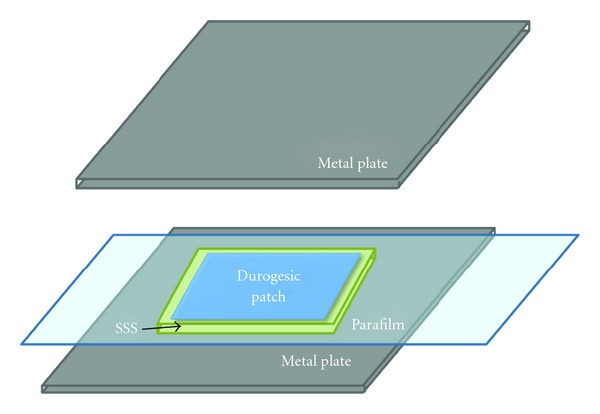
Illustration of the SSS method setup.

The SSS of the correlated patch size (5.25 cm^2^ for 12 *μ*g/hr patch or 31.5 cm^2^ for 75 *μ*g/hr patch) was prepared with three different moisture level (29, 57, 198 *μ*L cm^−2^) with pH 6.8 phosphate buffer and placed on a flat clean surface. The Durogesic patch was placed on the SSS at ambient temperature and humidity. Patch and SSS were placed on a piece of parafilm and covered with a flat metal plate on the top to avoid patch dislocation. The experimental setting is illustrated in Figure 1. The patch was moved to a new piece of wetted SSS after 1, 2, 3, 4, 6, and 24 hours and the old SSS was collected and soaked in HCl solution (pH1.0). The concentration of fentanyl in the solution was measured using isocratic reversed phase high-pressure liquid chromatography (HPLC). The experiments were performed in triplicates and the error bars were denoted by confidential interval. The fraction of drug release was calculated from the total amount of drug in the patch which was referred to the label claim [21].

### 2.3. Drug Release Test of Durogesic Patch Using USP Apparatus 5

Dissolution bath apparatus 2 with mini vessels (Sotax AT7 Smart, Sotax AG, Switzerland) was used to perform the release test for 12 *μ*g/hr patches. The patches were fixed in the so-called japaneese baskets and placed at the bottom of the vessel in 200 mL pH 6.8 phosphate buffer. While for 75 *μ*g/hr patches, dissolution bath apparatus 5 (Sotax AT7 Smart, Sotax AG, Switzerland) was used. The patches were glued to metal disks and placed at the bottom of the vessel in 900 mL pH 6.8 phosphate buffer with its release surface up. Both dissolution tests were performed with paddle speed of 50 rpm at 37°C. The concentration of fentanyl in the solution was measured using isocratic reversed phase liquid HPLC. The experiments were performed in duplicates and the error bar of each point was denoted by the confidential interval. The fraction of drug release was calculated from the total amount of drug in the patch which was referred to the label claim [21].

### 2.4. Analysis of Dissolution Curve

Similarity factor, *f*_2_, is one of the statistic tools to compare the dissolution profile and recommended by Polli and his colleagues [26]. It is also used by FDA and EMEA guidelines to compare dissolution curves for solid oral dosage forms [27, 28] and has been adopted by many studies to compare dissolution profile of different transdermal patch formulations [29–31]. Higher *f*_2_ value indicates higher similarity of two profiles, that is, two identical profiles gives *f*_2_ equals to 100. Two dissolution profiles are considered comparable when *f*_2_  is larger than 50. The formula of *f*_2_is shown as

(1) $$\begin{array}{c} f_{2}=50\times \log\left\{{\left[1+\left(1/P\right)\sum_{i=1}^{P}{\left(\mu_{ti}-\mu_{ri}\right)}^{2}\right]}^{-1/2}\times 100\right\} \end{array}$$

(see [32]), *P*  represents the number of time points for each measurement and *μ*_*i*_ is the fraction of release at time point *i*. Subscript *t* and *r* indicates test and reference, respectively. In this study, tests were results from USP apparatus 5 and SSS method, and the reference was the stated release profile.

## 3. Results and Discussion

### 3.1. Effect of the Amount of Moisture on the SSS on Release Rate

**Figure 2:**
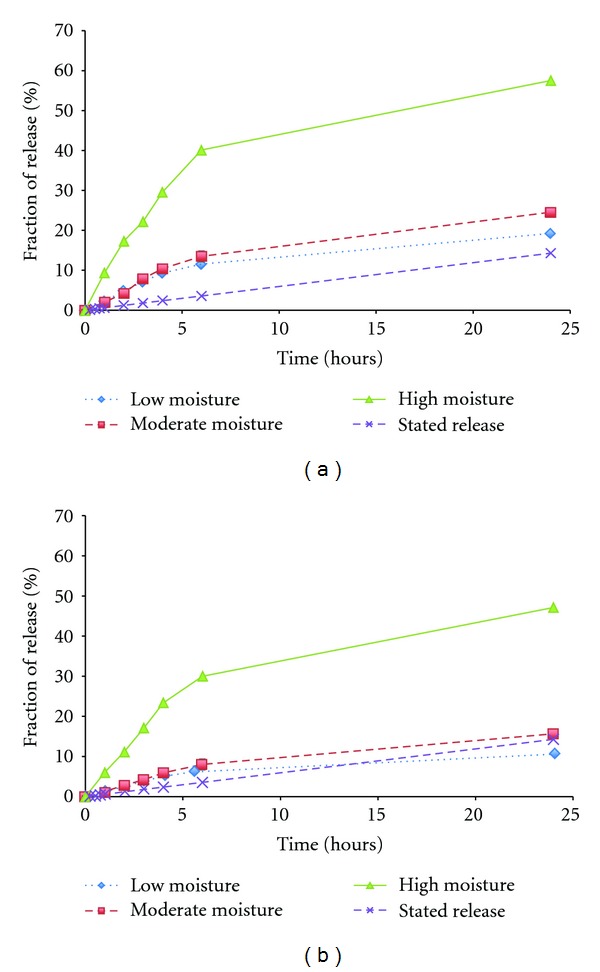
The effect of amount of moisture on the SSS on the drug release rate of Durogesic patches (12 *μ*g/hr (a); 75 *μ*g/hr (b)). The error bars denote the confidential interval (*n* = 3) and are shown where they exceed the dimensions of the symbols.

Generally, accumulated moisture between patch and skin is unavoidable and influences the rate of drug transport across the skin [20] and, therefore, drug release rates from the model patches were measured on the SSS with different moisture levels. The results showed that the drug release rate increased with increasing humidity level within 24 hours, see Figure 2. For both strengths of the patches, the release rates were similar under low and moderate moisture conditions but increased significantly under the high moisture condition. Release profiles obtained under low and moderate moisture on SSS were more similar to the stated clinical release of the patch. The release profile of larger patch had a higher correlation to the stated clinical release, which might result from the less edge effect comparing to smaller patch, that is, a smaller portion of the total amount of drug was released from the edge of the larger patch.

The results showed that a change of moisture level could cause the considerable change of the release profile and that the diffusion of fentanyl from the patch increased with the amount of accessible moisture. Therefore, the level of humidity on SSS should be carefully chosen to simulate the proper moisture between the patch and the skin. In this study, the moderate humidity, that is, 57 *μ*L cm^−2^, added to the SSS was the most suitable for Durogesic patch.

### 3.2. Comparison of an USP Method and the SSS Method

**Figure 3:**
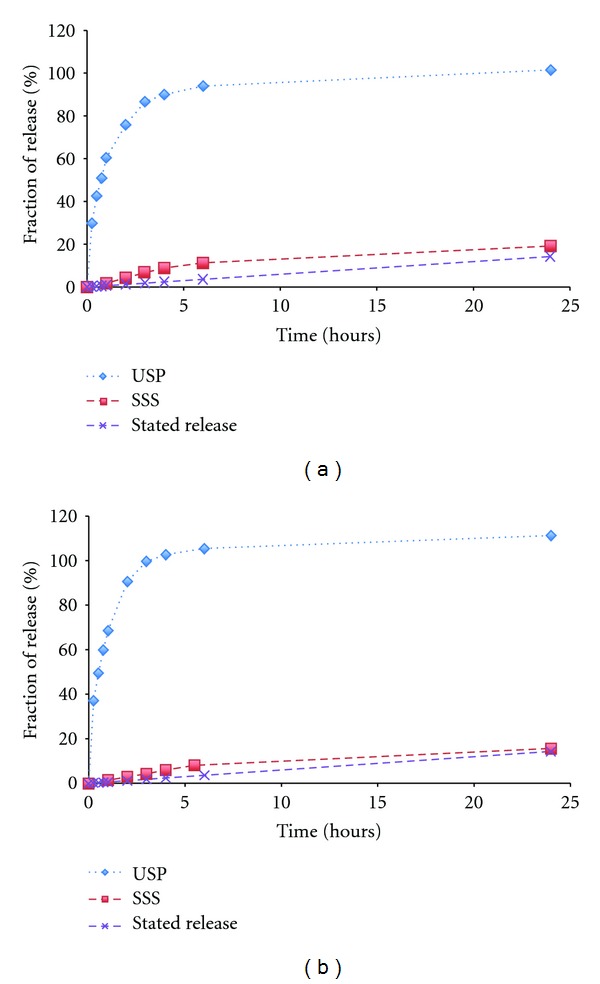
Comparison of the *in vitro* release profiles of 12 *μ*g/hr (a) and 75 *μ*g/hr (b) Durogesic patches using SSS and USP method with the stated release, error bars denote confidence intervals (*n* = 3) and are shown where they exceed the dimensions of the symbols.

As shown in Figure 3, Durogesic patch released more than 90% of fentanyl content within 5 hours using the USP method for both strengths, while the release profiles obtained by the SSS method with the moderate humidity on the SSS indicated a prolonged drug release. The release profiles were also compared with stated release of the patch, using similarity factor, as shown in Table 1. The results showed that the dissolution profile obtained from the SSS method was more comparable to the release claimed in the product label. In addition, the results correlates well to results reported in a previous study using the Franz cell method where the drug release rate varied between 1400 to 2600 ng cm^−2 ^h^−1^ [33].

Further, the drug release could be limited by a low dissolution rate of fentanyl in PH 6.8 and thus did not fit either the zero or first order release model [34]. Since the USP method provides sink condition, drug can be released with less “skin resistance.” This leads to a overestimation of the drug release from the patches where the drug diffusion mainly depends on skin permeation, thereby showing low correlation with the drug release profile with *in vivo* studies [13]. The results in this study indicate that the SSS method could imitate the skin resistance effect better than the USP method for Durogesic patches. The new method could therefore simulate the diffusion resistance and thus provide a significantly better estimation of *in vivo* performance than the USP method.

### 3.3. Limitations of the SSS Method and Further Work

The SSS method is an easy-implemented method developed for screening transdermal formulations in the early development. However, during the experiment, incomplete extraction of the drug from the SSS and drug release from the edges of the patch could be a possible source of errors in the measurement of drug release profiles. Fentanyl has a much higher solubility at lower pH [34] and the drug diffusion was regarded in equilibrium after extraction. Thus, nearly all amount of the drug should be released from the SSS and extraction should be considered to be complete. We found that a small amount of fentanyl was further released from the SSS after the first extraction. To minimize drug leaking out from the edge of the patch, the SSS was cut in the same size as the patch. Although those effects were insignificant, other error sources, such as evaporation of moisture, saturation on SSS, and temperature effect, could exist in the SSS method. Sealing the patch and SSS, replacing the SSS more frequently and testing under 32°C could help to reduce the potential errors. 

The scope of this study was to give a preliminary evaluation on the SSS method and a understanding of the effect of the amount of moisture on the drug release profiles of Durogesic patches. There are more interesting aspects that could be further investigated, such as estimation on the evaporation during testing, investigation of the saturation effect of the SSS, and assessment on other commercial transdermal products. 

## 4. Conclusions

A simple, low-cost, and selective method for measuring release rate of transdermal drug delivery system has been developed and evaluated. This method is based on a moisturized synthetic skin simulator (SSS), providing release resistance as well as a moisture and reservoir function for the drug released from the model patch. It was found that the drug release rate increased with increasing humidity level on SSS. This method showed a more valid way to measure the drug release profile than using the conventional *in vitro* release USP method. It could be particularly helpful in the work of screening various formulations in an early stage of research and development of transdermal drug delivery systems.

## Figures and Tables

**Table 1:** Similarity factors, *f*_2_, comparing stated release profile and release measured by USP apparatus 5 and SSS method.

	12 *μ*g/hr Durogesic patch		75 *μ*g/hr Durogesic patch	
	USP apparatus	SSS method	USP apparatus	SSS method
*f*_2_ value	4.46	63.89	1.54	77.01
